# HIV Pre-exposure Prophylaxis and Its Impact on the Gut Microbiome in Men Having Sex With Men

**DOI:** 10.3389/fmicb.2022.922887

**Published:** 2022-06-23

**Authors:** Nicola Luigi Bragazzi, Rola Khamisy-Farah, Christina Tsigalou, Naim Mahroum

**Affiliations:** ^1^Laboratory for Industrial and Applied Mathematics (LIAM), Department of Mathematics and Statistics, York University, Toronto, ON, Canada; ^2^Clalit Health Services, Akko, Israel; ^3^Azrieli Faculty of Medicine, Bar-Ilan University, Safed, Israel; ^4^Laboratory of Microbiology, Department of Medicine, Democritus University of Thrace, Alexandroupolis, Greece; ^5^International School of Medicine, Istanbul Medipol University, Istanbul, Turkey

**Keywords:** HIV/AIDS, prevention, prophylaxis, gut microbiome, men having sex with men

## Abstract

HIV/AIDS still imposes a high epidemiological and societal burden. Together with antiretroviral therapy, pre-exposure prophylaxis (PrEP) represents a fundamental tool in the fight against HIV/AIDS. PrEP is considered effective and safe, even though it may affect organs like the kidney, bone, and liver, as shown by randomized clinical trials (RCTs). These side effects may be mediated by alterations of the gut microbiome. Whilst the impact of the human rectal and vaginal microbiome on HIV prevention has been highly investigated among women, less is known about its effect among men having sex with men (MSM), a vulnerable population at high risk for HIV and disproportionately affected by HIV/AIDS. In the present paper, we will overview the effects of PrEP on the gut microbiota in MSM. Mining PubMed/MEDLINE, we identified three studies that have found significant changes affecting the gut microbiota. However, these shifts in the gut microbiome composition are variable, probably due to methodological differences, even though all studies reviewed in the present overview consistently report aberrations at the level of the gut microbiota. More data are needed, especially concerning the long-term side effects of PrEP: despite the studies included being a high-quality RCT, and two well-designed cross-sectional studies, evidence related to the impact of HIV PrEP on the gut microbiome in MSM is scarce and based on small populations. A better understanding of the interactions between the gut microbiota, sexual orientation/identity, and HIV prevention is expected to improve PrEP adherence and devise strategies to counteract PrEP-related side effects.

## HIV/Aids and Its Burden

Despite tremendous scientific and clinical advancements, HIV/AIDS still imposes a dramatically high epidemiological and societal burden. According to the “Global Burden of Disease” (GBD; GBD HIV Collaborators, 2017) Study, in the period from 1980 to 2017 (GBD HIV Collaborators, 2017; Pandey and Galvani, 2019), global HIV-related mortality peaked in 2006 with 1.95 million deaths and subsequently decreased to 0.95 million deaths, whereas new HIV diagnoses peaked in 1999 (with new 3.16 million cases) and gradually decreased to 1.94 million in 2017. This trend has been possible thanks to the introduction of effective antiretroviral medications, the scale-up and roll-out of which have significantly curbed HIV-related mortality, while, on the other hand, have increased the prevalence of people living with HIV (PLWH)—approximately, 36.8 million in 2017 [a similar figure in 2019 (GBD HIV Collaborators, 2019), 0.84 males per female, 0.99 male infections for every female infection, and 1.02 male deaths per female death]. HIV prevalence is particularly high in low-resource settings and in southern sub-Saharan Africa, with a highly variable coverage of antiretroviral therapy (ART).

Recently, a new technology has been introduced in the pharmacological armamentarium against HIV/AIDS: the so-called pre-exposure prophylaxis, or PrEP.

## HIV Pre-exposure Prophylaxis and Its Side Effects

Truvada^®^, a fixed-dose combination antiretroviral medication for HIV prevention (PrEP), was approved by the United States (US) Food and Drug Administration (FDA) in 2012. Its ingredients are tenofovir disoproxil fumarate (TDF)—a nucleotide analog reverse transcriptase (RT) inhibitor (NARTI/NRTI) – and emtricitabine (FTC)—a nucleoside analog reverse RT inhibitor (NtARTI/NtRTI). Descovy^®^, approved by the FDA in 2016, is a combination of emtricitabine and tenofovir alafenamide (Spinner et al., 2016).

PrEP, if taken once daily as prescribed, is highly effective in preventing HIV, curbing its risk by more than 90% in men having sex with men (MSM). Together with ART, treatment-as-prevention (TASP), and treat-and-test strategies, PrEP is a fundamental tool in the fight against HIV/AIDS. The roll-out of PrEP has, indeed, contributed to halving new HIV diagnoses in countries like Australia and Scotland (Grulich et al., 2018; Estcourt et al., 2021).

While Truvada^®^ and Descovy^®^ are taken orally, Apretude^®^ represents the third formulation of PrEP, which consists of the long-acting, injectable combination of cabotegravir (an integrase inhibitor) and rilpivirine (a non-nucleoside RT inhibitor, NNRTI), administered every 2 months by a specialized healthcare worker (Kim, 2021). It was recently approved by the FDA, in 2021.

However, despite its high effectiveness, PrEP may have some major side effects, affecting organs like the bone, kidney, and liver. Some randomized clinical trials (RCTs) have shown signs of kidney impairment (with the insurgence of renal tubular toxicity, tubular dysfunction, and phosphaturia; Schaefer et al., 2022), decreased bone mineral density (Spinelli et al., 2019; Baranek et al., 2020), and increased values of hepatic transaminases (Mandala et al., 2014). According to a recently published global PrEP user meta-analysis (Schaefer et al., 2022), pooling together 17 articles and synthesizing 11 RCTs, totaling 13,523 participants, PrEP use was associated with an increased risk of grade 1 and higher kidney adverse events (with an odds-ratio, OR, of 1.49 [95% confidence interval, CI 1.22–1.81]) and grade 2 and higher events (with an OR of 1.75 [95%CI 0.68–4.49]). Changes in bone mineral density are variable in the existing scholarly literature, with a substudy of the international, open-label PrEP demonstration project iPrEx-“open-label extension” (OLE) study estimating a 1.2 and 0.5% decrease in the spine and hip bone mineral density, respectively (Spinelli et al., 2019). A recently published systematic review and meta-analysis (Baranek et al., 2020), pooling together 25 studies, has computed a significant bone mineral density drop at the level of the lumbar spine (with a mean difference, MD, of −0.82% [95% from −1.28% to −0.37%]), and hip (with an MD of −0.81% [95%CI from −1.22% to −0.40%]). On the other hand, the use of PrEP was not associated with an increased risk of fracture. Concerning hepatic toxicity (Mandala et al., 2014), this can be mild to moderate, especially in individuals who are hepatitis B virus (HBV) surface antibody (HBsAb) positive or have underlying liver disease.

The cellular and molecular mechanisms of these adverse events are still poorly understood, and given the increasingly prominent role of PrEP in the fight against HIV/AIDS, it is of paramount importance to better understand them. Some scholars hypothesize that reduced bone mineral density may be due to changes in osteoblast gene expression or could occur *via* osteoprotegerin (OPG)/receptor activator of nuclear factor κΒ (NFκΒ; RANK) ligand (RANKL)/RANK system dysregulation (Brown et al., 2011; Delpino and Quarleri, 2020), whereas tubular injury may be mediated by the expression of the organic anion transporter 1 (OAT1) and the multidrug resistance protein 4 (MRP4) transporter (Kohler et al., 2011).

PrEP may result also in gastrointestinal side effects (especially within the first 3 months of treatment, known as “PrEP start-up syndrome”; Mugwanya and Baeten, 2016), and may alter the gut microbiome, which is functionally linked to the kidney, bone, and liver. Whilst the impact of the human (vaginal and rectal) microbiome on HIV prevention has been highly investigated and reviewed among women (Velloza and Heffron, 2017; Farcasanu and Kwon, 2018; Abdool Karim et al., 2019), less is known about its effect among MSM, despite the fact that this population is particularly at high risk for HIV and is disproportionately affected by HIV/AIDS. To the best of our knowledge, there are no comprehensive reviews covering this important topic. In the present paper, we will briefly overview the effects of the use of PrEP on the gut microbiota in MSM. For this purpose, we have mined PubMed/MEDLINE, the major biomedical database, searching for words such as “gut flora,” “gut microbial communities,” “gut microbiota,” “gut microbiome,” “intestine,” “rectal,” “anal,” “HIV,” “AIDS,” “men having sex with men,” “sexual identity,” “sexual orientation,” “tenofovir,” “emtricitabine,” and “pre-exposure prophylaxis.”

## The Gut Microbiome and Its Impact on Health and Disease

The gut microbiome is extremely heterogeneous and plastic and consists of a highly diverse population of bacteria that can have both beneficial and detrimental impacts on human health (Fan and Pedersen, 2021). It is composed of more than 1,200 species of bacteria (Rinninella et al., 2019), including *Bacteroides*, *Actinomycetes*, *Firmicutes*, *Proteobacteria*, and *Verrucomicrobia*. It plays different functions, ranging from nutrient absorption (in particular, micro-nutrients), and processing to metabolic homeostasis (including favoring insulin sensitivity), and fine-tuning of the immune system, protecting especially newborns from respiratory and intestinal infections and pathogen invasion (Flint et al., 2012). It can also provide the individual with sources of energy, by fermenting and processing short-chain fatty acids (SCFAs), like butyrate, acetate, and propionate (Valdes et al., 2018).

Aberrations at the level of the gut microbiota and enteric dysbiosis have been linked with several disorders, like autoimmune diseases, such as rheumatoid arthritis (RA; Bodkhe et al., 2019), liver disease (like non-alcoholic fatty liver disease or NAFLD; Wang et al., 2021), overweight and obesity, type 1 and 2 diabetes, malnutrition, and other metabolic/nutritional impairments, malignancies (especially colon cancer; Tortora et al., 2022), irritable bowel syndrome (IBS), inflammatory bowel disease (IBD; Glassner et al., 2020), and neurological diseases (Parkinson’s disease, transient cerebral ischemia, and ischemic stroke; Pluta et al., 2021; Romano et al., 2021), among others.

Gut microbiome-axes have also been demonstrated for the numerous organs impacted by PreP. The gut microbiota is dynamically interconnected with the kidney. In individuals with chronic kidney disease (CKD), it may be impaired and produce several uremic toxins and solutes, like p-cresyl sulfate (PCS), trimethylamine (TMA) N-oxide (TMAO), and indoxyl sulfate. In turn, increased urea levels result in gut microbiome alterations. The release of uremic toxic metabolites may lead to renal anemia, asthenia, pruritus, impaired mineral bone density, cardiovascular, and neurological disorders, which are commonly observed in CKD patients as well as in subjects with acute kidney injury (AKI), IgA nephropathy, nephrolithiasis, hypertension, or those needing hemodialysis/peritoneal dialysis (Chen et al., 2019; Hobby et al., 2019; Stavropoulou et al., 2021). Gut microbiota overgrowth or change in profile composition may result in alterations in calcium, vitamin K, and vitamin B levels, as well as in vitamin D absorption and metabolism, leading to fractures and osteoporosis (Ding et al., 2020). Cellular and molecular mechanisms include modulation of insulin-like growth factor (IGF)-1-related cascades. IGF-1 is known to fine-tune the bone cells’ differentiation into osteoblasts, osteoclasts, and chondrocytes (Pacifici, 2018). The gut microbiota can also affect the concentrations of vitamin D metabolites, steroid hormones, and the parathyroid hormone (PTH; Pacifici, 2018). A new, high interdisciplinary field is emerging, termed “osteomicrobiology,” to underline the strong links and connections between bone and microbes (Bhardwaj et al., 2022). The gut microbiome also impacts liver functioning. Gut microbiota overgrowth and, in particular, small intestinal bacterial overgrowth (SIBO) of Gram-negative pathogens contribute to reducing overall microbial diversity and to tissue damage, by the production and release of bacterial 16sDNA, ethanol, toxic metabolites, and endotoxins (like TMAO and LPS), which inhibit cholesterol conversion into bile acids, alter intestinal permeability, promote a pro-inflammatory *milieu*, modulate apoptotic cascades and exacerbate the underlying liver disease (Zhou et al., 2021). Impaired fermentation and processing of SCFAs result in decreased stimulation of gut endocrine cells and reduced secretion of glucagon-like peptide 1 (GLP-1) with a subsequent drop in hepatic fatty acid β-oxidation and lipogenesis. Also, PPARα-mediated β-oxidation of fatty acids is altered, leading to a worsening of hepatosteatosis and liver disease. Moreover, aberrant gut microbiota leads to the activation of hepatic stellate cells (HSCs) *via* the intrahepatic T-cell receptor (TCR) immune repertoire (IR; TCR-IR; Liang et al., 2020). This results in the shift from a quiescent to an active, proliferative, migratory, and fibrogenic phenotype, transforming the cell into a myofibroblast, contributing to liver fibrogenesis.

## The Gut Microbiome and HIV/AIDS

Among the different diseases, changes in the human microbiome composition have been associated with HIV serostatus (Noguera-Julian et al., 2016; Tuddenham et al., 2020; Zhou et al., 2020), with a few exceptions (Li et al., 2019). Shifts in microbial composition have been linked as well with sexual orientation/sexual identity (Noguera-Julian et al., 2016; Li et al., 2019; Tuddenham et al., 2020; Zhou et al., 2020), as shown also by animal models (Li et al., 2019). Li et al. (2019) transplanted feces from HIV-negative men having sex with women (MSW) and MSM, as well as from HIV-positive untreated MSM to gnotobiotic mice. After the transplant, the microbiomes of MSM and MSW conserved distinct compositions in mice. From an immunological standpoint, HIV-negative MSM donors exhibited higher frequencies of blood CD38+ HLA-DR+ and CD103+ T-cells whereas their fecal recipients had higher frequencies of gut CD69+ and CD103+ T-cells. Probably due to the small sample size employed, the authors failed to detect statistically significant differences induced by HIV serostatus both at the level of the microbiome and immunologically. On the other hand, after infecting primary human *lamina propria* cells treated with fecal microbiota, gut flora from MSM caused higher levels of HIV replication. Among humans, HIV-seropositive individuals and MSM consistently report alterations in the gut flora, affecting, for instance, *Bacteroides caccae*, *Bacteroides ovatus*, *Bacteroides uniformis*, and *Prevotella stercorea*, with markedly reduced richness and alpha diversity of the gut microbiota. This can be accompanied by profound metabolic changes involving several functional pathways (such as those regulating carbohydrate, lipid, and amino acid metabolism), according to a recently published systematic review and meta-analysis of 12 studies (Zhou et al., 2020).

Further, the gut microbiome may increase the risk of contracting HIV (Coleman et al., 2020) *via* increased expression of integrin and chemokine receptors on T-cells, especially at the level of the colon. Furthermore, it may influence HIV transmission (Coleman et al., 2020), favoring HIV persistence (Koay et al., 2018) through immune activation and systemic inflammation, and impacting the progression from acute HIV infection (AHI; Sortino et al., 2019) to AIDS (Sortino et al., 2019), with microbiota diversity correlating with CD4+ T-cell diversity (Koay et al., 2018; Sortino et al., 2019). As the disease progresses, an increase in pathogenic species, including *Proteobacteria*, and a decrease in protective ones (like *Bacteroides*, *Lactobacillus*, and *Bifidobacterium*) are observed (Ling et al., 2016; Ribeiro et al., 2017; Vujkovic-Cvijin et al., 2020). The intestinal microbiome plays a key role also in the pathogenesis of HIV/AIDS- and age-related comorbidities, like cardiovascular disease, stroke, malignancy, long-bone fractures, and renal impairment/dysfunction (Sortino et al., 2019). Moreover, the gut microbiota has effects on HIV drug metabolism, pharmacokinetics, and effectiveness (Imahashi et al., 2021).

## The Gut Microbiome and HIV Pre-exposure Prophylaxis

Few studies have investigated the impact of HIV PrEP on the gut microbiome, by conducting metagenomic analyses and next-generation sequencing (NGS) of the bacterial 16S/16S-23S ribosomal RNA (rRNA) gene(s) in highly adherent PrEP users to assess the relative abundance of family and genus and potential changes/shifts in the enteric microbiome. Features of these studies are summarized in Table 1.

**Table 1:** Major features of studies overviewed in the present mini-review.

Study	Sample size	Study design	Specimen	Microbiome profiling technique	Statistical analysis	Age	Race/ethnicity	Factors potentially affecting the gut microbiota	Sexual behavior	PrEP treatment length	Adherence to PrEP	Increased gut flora	Decreased gut flora	Alpha diversity	Beta diversity	Overall diversity and composition
Dubé et al., 2018	8	Sub-study from the RCT NCT01761643 (cohort “California Collaborative Treatment Group study 595,” CCTG-595)	Self-collected rectal swab	Short-read sequencing of bacterial 16S rRNA gene V4 region and long-read sequencing of bacterial 16S–23S rRNA gene V4 region	Permutation tests and PCA clustering analysis	34.8 ± 9.1 (range 22–52) years	Half white, one Asian, one black, and two “of multiple races”	6/8 reporting rectal douching 6/8 reporting any drug use 3/8 reporting drug abuse	NA	48–72 weeks (72 weeks in 5 participants, 48 weeks in 2 subjects, and 60 weeks in 1 participant)	Very good or excellent in 100% of the participants	*Erysipelotrichaceae* (*Catenibacterium mitsuokai*, *Holdemanella biformis*, and *Turicibacter sanguinis*)	*Streptococcaceae* (*Streptococcus agalactiae*, *Streptococcus oralis*, and *S. mitis*)	No changes	β diversity between each participant’s pre and post-PrEP specimens was smaller than the β diversity of pre-PrEP specimen pairs of different individuals	No changes
Fulcher et al., 2019	74 (37 on PrEP versus 37 off PrEP)	Cross-sectional, matched case–control study, with participants selected from the ongoing cohort mSTUDY	Rectal swabs collected under direct mucosal visualization *via* anoscopy without preparatory enema	Sequencing of the V4 region of the 16S rRNA gene	Zero-inflated negative binomial models, multinomial LASSO as confirmatory analysis	30.1 ± 6.7 (median 29) years for the control and 29.8 ± 6.5 (median 28) years for the PrEP users’ group	56.8% black non-Hispanic, 32.4% Hispanic, 10.8% other non-Hispanic	26/37 reporting marjuana use 7/37 reporting methamphetamine use 10/37 reporting tobacco use 28/37 reporting binge alcohol use 4/37 reporting antibiotic use in the past month 35.1% obese	45.9% recent receptive anal intercourse (2.2 ± 4.5) *per* month)	41 weeks (IQR 14–59 weeks)	Very good or excellent in 78% of the participants	*Streptococcus*, *Mitsuokella*, and *Fusobacterium*	*Escherichia* and *Shigella*	No changes	NA	No changes
Perler et al., 2021	27: 14 taking PrEP	Cross-sectional, case–control study	Self-collected rectal swabs	Sequencing of the V4 region of the 16S rRNA gene	Linear regressions, linear discriminant analysis, Bray–Curtis dissimilarity analysis	36 years (on PrEP), 29 years off PrEP	NA	NA	8.43 partners *per* month	171 weeks (3.3 years)	NA	*Catenibacterium* and *Prevotella* 2	*Pasteurellaceae*, *Corynebacteriaceae*, *Clostridiales* Family XI (at the family level), and *Finegoldia* and *Corynebacterium* 1 (at the genus level)	No changes	Significant changes in the PrEP group	No changes

Dubé et al. (2018) were the first to detect and report alterations in the microbiome composition, with *Erysipelotrichaceae* (in particular, *Catenibacterium mitsuokai*, *Holdemanella biformis*, and *Turicibacter sanguinis*) and *Streptococcaceae* (especially, *Streptococcus agalactiae*, *Streptococcus oralis*, and *Streptococcus mitis*) being increased [by a mean log_2_ (fold change) of 2.1, from 0.79 to 3.3%] and being decreased [by a mean log_2_ (fold change) of −3.3, from 12.0 to 1.2%], respectively, already after 48 weeks of PrEP administration. No correlation with PrEP duration, antiretroviral levels, race, age, drug use, or abuse could be found. The authors hypothesized that changes in the gut microbiome may be linked with serious long-term side events, like colorectal malignancy, metabolic, and cardiovascular disease. However, at the moment, this remains a hypothesis not supported by clinical data, that, anyway, warrants further investigations, according to the same authors.

Different findings were reported by Fulcher et al. (2019), who performed a cross-sectional study, comparing off versus on PrEP users, matched using propensity score 1:1 matching and performing logistic regression based on several covariates. These included age, race/ethnicity, BMI, smoking, alcohol and drug use (methamphetamine, and marijuana), and antibiotic use in the past month (single-dose azithromycin, ceftriaxone, or doxycycline), and sexual practices (recent receptive anal intercourse in the last 7 days and number of acts in the last month). Differently from Dubé et al. (2018) and Fulcher et al. (2019) found that the use of PrEP significantly correlated with an increase in *Streptococcus*. Other pathogens the abundance of which was increased were *Mitsuokella*, and *Fusobacterium*, while a decrease in *Escherichia* and *Shigella* abundance was observed. Of note, increased *Fusobacterium* was associated with increasing exposure to tenofovir, again differently from Dubé et al. (2018), who did not observe any correlation with antiretroviral concentrations. Similarly to Dubé et al. (2018) also Fulcher et al. (2019) warned about the potential long-term side-effects of oral PrEP: indeed, in the literature, increased *Fusobacterium* has been associated with inflammatory conditions, IBD, and colorectal cancer.

Finally, Perler et al. (2021) compared on and off PrEP individuals. In the group of PrEP users, the authors detected a statistically significant decrease in *Pasteurellaceae*, *Corynebacteriaceae*, *Clostridiales* Family XI (at the family level), and *Finegoldia* and *Corynebacterium* 1 (at the genus level), and an increase in *Catenibacterium* and *Prevotella* 2, while no changes were observed at the phylum level. Noteworthy, the increase in *Catenibacterium* had been previously reported by Dubé et al. (2018). *Prevotella* 9 was slightly higher in the PrEP user’s group, but not in a statistically significant way. Moreover, this group had a higher number of male receptive anal sex partners, which correlated with the relative abundance of *Prevotella* 2, but not of *Finegoldia* or *Streptococcus*. No changes/shifts in *Streptococcaceae* and *Erysipelotrichaceae* could be observed, differently from previously published studies (Dubé et al., 2018; Fulcher et al., 2019). Of note, no differences could be detected in terms of human behaviors and lifestyles, like drugs/antibiotics exposure, sexual practices (anilingus), hygiene (rectal douching/enema), use of stool softeners, probiotic supplementations, use of lubricants, or use of saliva as a lubricant, and gastrointestinal symptoms, among others. Interestingly, sexually transmitted infections (STIs) were not found to impact the gut microbiome composition. Finally, alpha diversity did not change (as in previous studies; Dubé et al., 2018; Fulcher et al., 2019), differently from beta diversity. In line with the other studies overviewed in the present review (Dubé et al., 2018; Fulcher et al., 2019), the authors concluded that increased pathogens may lead to long-term periodontal disease, RA, insulin resistance, morbid obesity and metabolic impairments, and cardiovascular disease.

While all the studies analyzed consistently report changes and shifts in the gut microbiota, there are differences at the phylum, family, and/or genus level. This could be due to: (i) the different study design (cross-sectional versus longitudinal, case–control versus RCT), (ii) the sample size employed, (iii) the microbiome analysis methods and techniques used for specimen collection (rectal swab versus mucosal biopsy; Araújo-Pérez et al., 2012), and bioinformatics/statistical analysis, and (iv) the period of treatment (from a few months to some years, short- versus long-term). Moreover, further information should be collected about lifestyles, including dietary intake, exercise/physical activity, and sexual practices, which are known to modulate the gut microbiome composition. For example, the use of lubricants could have an effect on gut flora. Haaland et al. (2018) investigated whether the rectal application of a hyperosmolar lubricant impacted tenofovir drug tissue concentration in the rectum and related secretions and the composition of the gut microbiome. The authors found no effects on mucosal PrEP concentration but detected an impact of the use of lubricant on the gut microbiota, with decreased *Bacteroides*, increased concentrations of *Prevotella* (statistically borderline significant), and increased microbial diversity. A statistically significant interaction between the use of PrEP and lubricant was found for the relative abundance of the genus *Alicyclobacillus*. Of note, not all the studies analyzed, have adjusted for confounding factors.

## Conclusion and Future Prospects

As stated by Hughes et al. (2020), PrEP represents a unique opportunity to study the effects of Truvada^®^ and Descovy^®^ for PrEP on the gut microbiota without the interference of HIV, other drugs, and/or HIV/AIDS-related co-morbidities, as well as other confounding factors. These drugs are generally considered effective and safe. However, the study by Hughes and collaborators (Hughes et al., 2020) and the studies retained in the current review (Araújo-Pérez et al., 2012; Haaland et al., 2018; Hughes et al., 2020) have shown that they may alter and impair the gut microbiota, and repress the transcription of several nuclear transcription factors, inhibit the anti-inflammatory function of mucosal epithelial cells, by favoring a pro-inflammatory milieu, and promote gene signatures related to increased cell viability and proliferation as well as increase the abundance of pathogenic microbes, potentially linked to serious conditions and diseases. However, more data are urgently needed, especially concerning the long-term side effects of PrEP: despite the fact that the studies included in the present overview are a high-quality RCT, and two well-designed cross-sectional studies, evidence related to the impact of HIV PrEP on the gut microbiome in MSMs is still scarce and based on small populations. A better understanding of the complex, nonlinear interactions between the gut microbiota, sexual orientation/identity, and HIV prevention is expected to significantly improve PrEP adherence and potentially devise interventional strategies to counteract PrEP-related side effects.

## Author Contributions

All authors listed have made a substantial, direct, and intellectual contribution to the work and approved it for publication.

## Conflict of Interest

The authors declare that the research was conducted in the absence of any commercial or financial relationships that could be construed as a potential conflict of interest.

## Publisher’s Note

All claims expressed in this article are solely those of the authors and do not necessarily represent those of their affiliated organizations, or those of the publisher, the editors and the reviewers. Any product that may be evaluated in this article, or claim that may be made by its manufacturer, is not guaranteed or endorsed by the publisher.
